# The *Shigella* siphophage Sf11 tail structure and host attachment mechanism

**DOI:** 10.1128/jvi.01367-25

**Published:** 2025-11-19

**Authors:** Sundharraman Subramanian, John A. Dover, Kristin N. Parent

**Affiliations:** 1 Department of Biochemistry and Molecular Biology, Michigan State University 123744 , East Lansing, Michigan, USA; University of Kentucky College of Medicine, Lexington, Kentucky, USA

**Keywords:** bacteriophage, cryo-EM structure, phage entry, tails, *Shigella *phage, siphophage

## Abstract

A paucity of reports is available describing the structures of *Shigella* phages, and these have focused to date on a few short, non-contractile podophages and one long contractile myophage. Here, we report the cryo-EM structure of a *Shigella* siphophage, where we can visualize the capsid and surface decoration proteins and many components of the tail, including the Tail Tube Protein (TTP), Distal Tail Protein (DTP), Baseplate Hub Proteins (BHUB1 & BHUB2), and the Tape Measure Protein (TMP). The tail is also decorated with six copies of a trimeric tailspike protein that is similar to Sf6-like and P22-like phages. We used mass spectrometry to confirm the identity of the proteins in the mature virion and present atomic models for the majority of these phage proteins. In addition, host range studies show clearly that these tailspike appendages have a homologous function to those in the Sf6-like and P22-like phages in recognizing the O-antigen on the host lipopolysaccharide (LPS).

**IMPORTANCE:**

Few *Shigella* phages have been studied structurally to date. By characterizing phage Sf11, we see evidence for a tail adaptor domain that is used for decorating the siphophage tail tip with enzymatic, P22-like tailspike proteins. This is important for both understanding the evolutionary relationships among *Shigella* phages and also could be exploited as a type of protein scaffolding for creating designer phages for therapeutic and/or industrial purposes.

## INTRODUCTION

Viruses that infect bacteria, called bacteriophages or phages, are highly abundant, with estimates on the order of ~10^31^ particles in the biosphere. Most phages contain dsDNA genomes contained within protein shells called capsids and tails that are responsible for host recognition and attachment. Originally, bacteriophages were grouped into families based entirely on the tail morphology, and these included the siphophages (long, non-contractile tails) (1–3), myophages (long contractile tails) (4), and podophages (short, non-contractile tails) (5). Although the International Committee on the Taxonomy of Viruses (ICTV) has recently steered away from these older classifications, they are still useful descriptions as virus structure is often highly conserved, even in the absence of sequence similarity.

Historically, siphophages have not been well described structurally. However, in the last few years, there has been a rapid increase in structural studies of siphophage tails and/or entire virions as cryo-electron microscopy has evolved. Some examples include lactococcal phage p2 (6), marine *Roseobacter* phage R4C (7), *Salmonella* phage χ (8), *Staphylococcus* phage 80α (9), and *Escherichia coli* phage T5 (10). Most siphophage tails have the same core building blocks (for review, see, [1–3, 11]). These include the tail completion protein, tail tube protein, neck protein, and tape measure proteins. The distal tip of siphophage tails is responsible for host recognition and attachment. Tail tips can be highly diverse in complexity, with many copies of the receptor binding proteins, and can vary drastically according to species. However, most, if not all, have the minimum components of Tal (tail-associated lysozyme) and DTP (distal tail protein).

There have been relatively few characterized *Shigella* phages (12) and studies on *Shigella* phages in general. The most well-studied *Shigella* phage is Sf6, a podophage with a comprehensive body of work regarding structure (13–15), biochemistry (16, 17), and genetics (18, 19). Research on Sf6 has been important in demonstrating a fundamental shift in the way we think of phage:host interactions, as it was shown that Sf6 has an innate ability to utilize more than one receptor type (12, 16, 17, 20). By contrast, the old canon of thought was that phages are highly specific and only utilize single, highly specific receptors. We sought to investigate whether innate multiple receptor usage was common among *Shigella* phages or specific to Sf6. In over 100 years of phage biological studies, there were fewer than 40 *Shigella* phages reported in the literature. This expanded greatly beginning in 2016 when the Parent lab began “hunting” for *Shigella* phages from a wide variety of environmental samples to understand host range infection profiles among *Shigella* phages (21–23). As a result of this work, structures of a few other *Shigella* phages have been described, such as the N4-like podophages Moo19 and B2 (24), podophage HRP29 (25), and myophage Sf14 (26). However, no structures of *Shigella* siphophages have been published to date.

Here, we use cryo-electron microscopy (cryo-EM) and receptor binding studies to describe the tail complex of *Shigella* siphophage Sf11. We resolved the entire virion at high resolution, where we were able to fully model the capsid, including the major capsid and head decoration proteins, and the portal complex. In addition, we were able to model the tail apparatus and tail tip. We confirmed the presence of virion-associated proteins with mass spectrometry, including some that were not resolved in the cryo-EM reconstruction. The core proteins in the tail are conserved and consistent with siphophages observed in other species. However, we show that an adaptor domain on the DTP is utilized to decorate the long non-contractile tail with Sf6-like tailspikes at the distal tip. Receptor binding studies show that this phage utilizes the O-antigen of lipopolysaccharide (LPS) as a primary receptor, similarly to its distant podophage relatives. Negative stain electron microscopy shows that infection is not localized to a specific feature (e.g., such as the cell poles, etc). The discovery of Sf6-like tailspike proteins spanning diverse podophages like Sf6, Moo19, B2, and the siphophage Sf11 suggests evolutionary relationships among tails of *Shigella* phages.

## RESULTS AND DISCUSSION

### Virion structure

**Fig 1:**
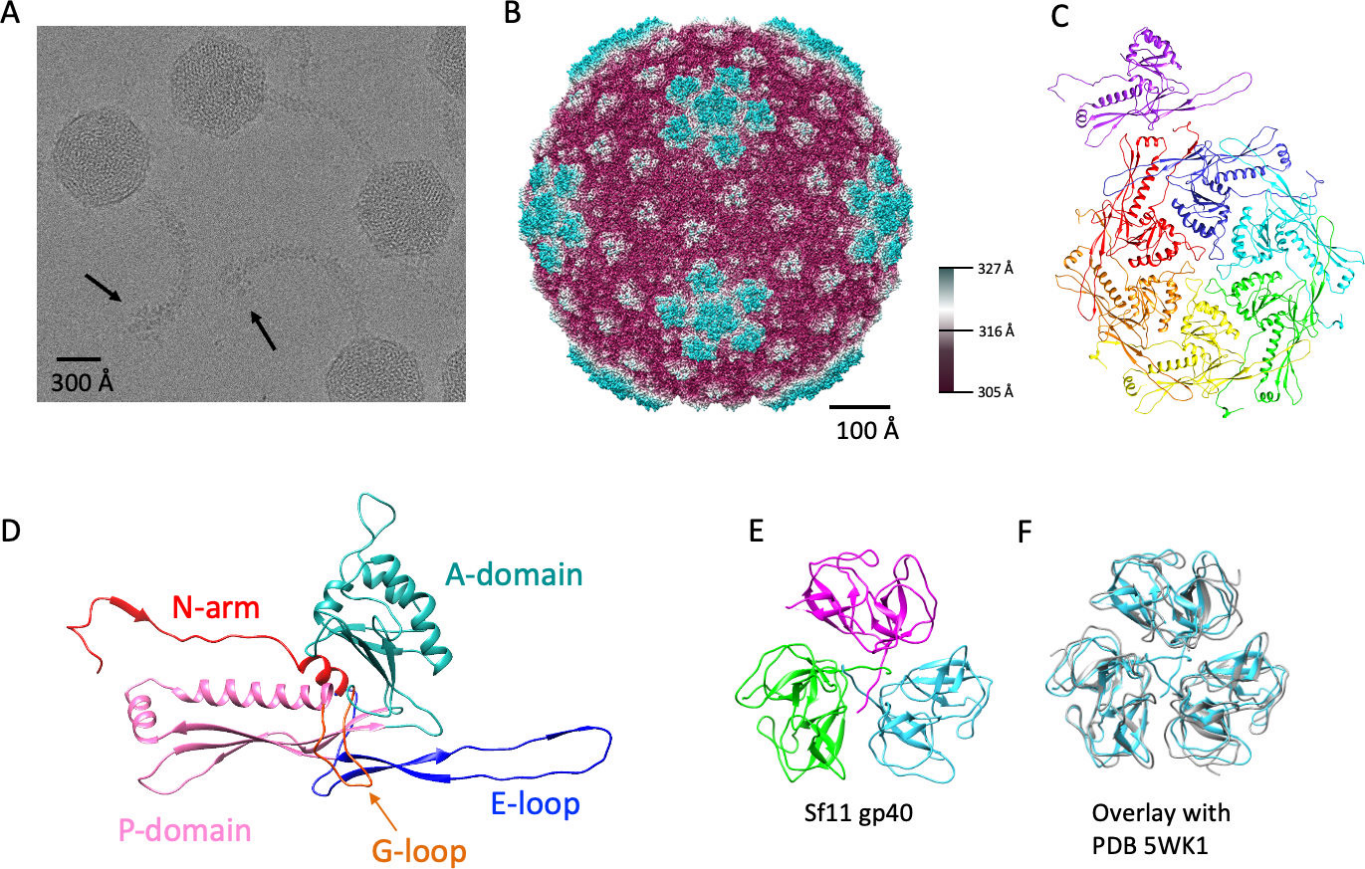
Cryo-EM structure of Sf11 capsid. (**A**) Representative cryo-EM micrograph of mature Sf11 virions. Black arrows point out tail spike density in the tail tip. (**B**) Surface rendering of the Sf11 icosahedral capsid color-coded by radius. (**C**) Asymmetric unit of the major capsid protein, with the individual chains rainbow colored. (**D**) HK97-like fold of a monomer of the major capsid protein color-coded by domain. (**E**) Asymmetric unit of the decoration protein (gp40) with individual chains colored in cyan, magenta, and green. (**F**) Alignment of Sf11 gp40 (gray) with the decoration protein from marine phage TW1 (PDB ID 5WK1) shown in cyan. Alignment was performed using the MatchMaker tool in UCSF Chimera (27).

*Shigella* phage Sf11 was isolated and briefly described previously using negative stain electron microscopy to confirm siphophage morphology (21). Here, we investigated the structure of the entire virion using high-resolution cryo-electron microscopy to visualize the entire virion in greater detail. See Fig. S1 and S2 for the cryo-EM processing workflow for all structures. The capsids have clear icosahedral symmetry and long, flexible tails, with decorated ends (Fig. 1A). We used mass spectrometry to identify proteins associated with a purified, high-titer Sf11 stock to determine which gene products were part of mature virions. Phage proteins listed in Table 1 were positively identified from trypsin-digested fragments of an excised SDS-PAGE gel band with over 95% confidence using Michigan State University’s core proteomics facility.

**TABLE 1:** Mass spectrometry results of Sf11 mature virions^*a*^

Protein annotation		Gene product	MW (kDa)	Total residues (residues modeled)	Copies per virion
Major capsid protein		**gp39**	**40**	356 (46–355)	415
Portal protein		gp42	52	470 (29–410)	12
Head morphogenesis protein		gp62	36	316 (N/A^*b*^)	??^*c*^
Baseplate hub 1 protein		**gp16**	**18**	156 (1–154)	3
Baseplate hub 2 protein		**gp14**	**92**	828 (81–827)	3
Tailspike protein		**gp13**	**62**	585 (4–59)	18
Tape measure protein		**gp18**	**82**	780 (749–780)	3
Decoration protein		**gp40**	16	155 (2–153)	415
Distal tail protein		**gp17**	**50**	457 (3–165)	6
Tail tube protein		gp24	25	238 (7–161)	6
Hypothetical protein		gp52	13	N/A	??
Head morphogenesis protein		gp62	36	N/A	??
Hypothetical protein		gp64	52	N/A	??
Hypothetical protein		gp68	30	N/A	??
Hypothetical protein		gp77	13	N/A	??
HK97 gp10 family protein		gp78	14	N/A	??

^*a*^ Bold font means observed in reconstruction.

^*b*^ N/A indicates not applicable.

^*c*^ ?? means we are not able to estimate, given the currently available data.

Next, we performed a reconstruction of the capsid imposing icosahedral (532) symmetry to 3.4 Å resolution. Sf11 has a ~50 kbp genome (Genbank accession number MF158038) encapsidated in an icosahedral shell formed by 415 copies of the major capsid protein in a typical T = 7 geometry (Fig. 1B and C). The major capsid protein (gp39) adopts the classical HK97-like fold adorned by most, if not all, dsDNA-tailed bacteriophages (28). The Sf11 major capsid protein model derived from our cryo-EM data contains many of the hallmarks of the HK97-like fold, including the P domain, E-loop, G-loop, and A domain (Fig. 1D). We were able to model residues 46–355 but could not resolve the full-length N-terminal “N-arm.” Mass spectrometry data of mature Sf11 virions (Table 1) showed nearly complete coverage of the mature major capsid protein spanning residues 40–356. Therefore, it is likely that the N-arm is cleaved during maturation, as is observed in phages such as HK97, T5, and others (29, 30). The copy number of each protein per virion and residues modeled is listed in Table 1 for all Sf11 structures shown.

Like many phages, the Sf11 capsid also has several copies of a surface-bound auxiliary protein (gp40) called a decoration, or “cement,” protein. Decorative proteins are often used to stabilize phage capsids and take on a few distinct folds (31). Visual inspection of the Sf11 decoration protein (gp40) shows that it forms a homotrimer (Fig. 1E) and is structurally similar to the β-tulip family of decoration proteins, such as phage λ’s gpD (32) and gp87 of the hyperthermophilic phage P74-26 (33), despite no recognizable sequence homology. A DALI search (34) indicates the best structural equivalent is from marine phage TW1 (PDB 5WK1) at 26% sequence similarity, a root mean square deviation (R.M.S.D.) of 1.6 Å for Cα atoms, and a Z score of 20.0. See Fig. 1F for an alignment showing structural homology. Furthermore, Sf11’s gp40 displays the same capsid binding occupancy as phage λ, as it binds the capsid at all of the icosahedral threefold symmetry sites. However, unlike phage λ, the N-terminus of gp40 does not appear to be the stabilizing binding force cementing the decoration protein to the capsid (35), as long extensions of the N-terminus of gp40 were not visible in binding to the capsid in the cryo-EM density map of Sf11.

### Portal and tail structures

**Fig 2:**
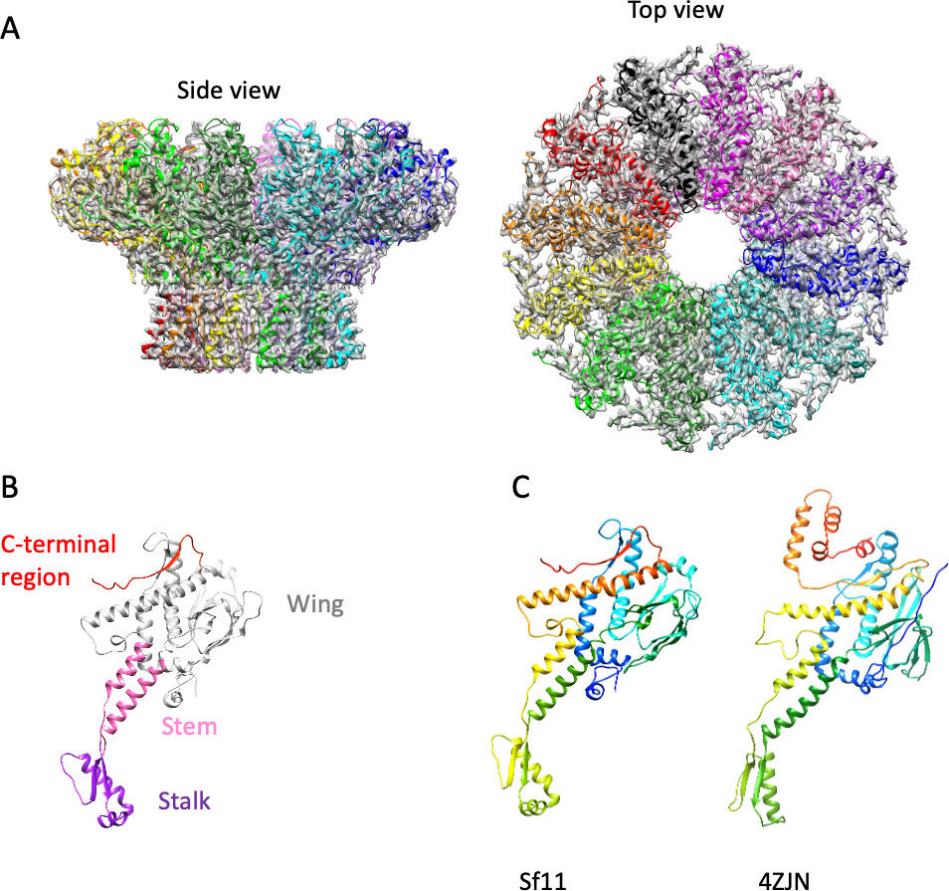
Cryo-EM reconstruction of the portal complex. (**A**) Side and top views of the portal dodecamer. Atomic models of the 12 subunits are docked into the cryo-EM density map, and each chain is colored individually. (**B**) Monomer view of the portal color-coded by functional domain. (**C**) Side-by-side comparison to the closest homolog, phage G20C portal (PDB ID 4ZJN). Proteins are color-coded in a rainbow scheme from the N-terminus (blue) to the C-terminus (red).

We performed a focused reconstruction of the capsid at the unique vertex that binds to the tail. First, we aligned the capsid using EMD-8867, which is the portal and neck of marine phage TW1 (36). We then relaxed the imposed icosahedral symmetry, recentered the particles to focus on the neck region, and applied C1, C3, and C6 symmetry, individually. Only C6 symmetry improved the refinement. We then further restricted the box size and performed 3D classification. We picked two classes that showed a clearly defined neck region, then applied C12 symmetry for an additional round of refinement, applying both local CTF correction and C12 symmetry. We were able to solve the portal complex to 2.35 Å (Fig. S2). ModelAngelo (37) was then used to refine the portal structure *de novo* (Fig. 2B). The portal of Sf11 is a dodecameric structure like the vast majority of phage portals (38) (Fig. 2A). The structure of the portal protein has domain features in common with many phages including obvious stalk, stem, and wing domains (Fig. 2B). It is unclear if the Sf11 portal has an extended C-terminal barrel such as phage P22 (39), as we could only model residues 29–410 of a 470 amino acid protein. However, prediction programs such as HHPred and AlphaFold 3 did not predict helical features in the C-terminus (data not shown). A DALI search shows that the closest structural homolog is PDB ID 4ZJN, a portal protein in bacteriophage G20 (40), with a Cα R.M.S.D. of 3.8 Å over 313 residues and a Z score of 20.2 (Fig. 2C).

Unfortunately, despite our best efforts, we could not resolve the rest of the neck region. We relaxed the C12 symmetry in the region adjoining our well-aligned portals and tried applying C1, C3, C5, and C6 symmetry individually. No improvement in the neck region was observed, and we believe this connector region is a bit flexible, inhibiting further refinement. Furthermore, we analyzed the remaining proteins detected in our mass spectrometry data that were not accounted for in our density maps (Table 1). This includes four proteins annotated as “hypothetical,” one annotated as a head morphogenesis protein, and one designated as an HK97 gp10 family protein. Despite using BlastP, AlphaFold 3, and DALI to analyze sequences and predicted protein folds, none had significant homology to any known neck or collar region in tailed phage. Therefore, we cannot determine which proteins comprise the neck region of Sf11 at this time.

**Fig 3:**
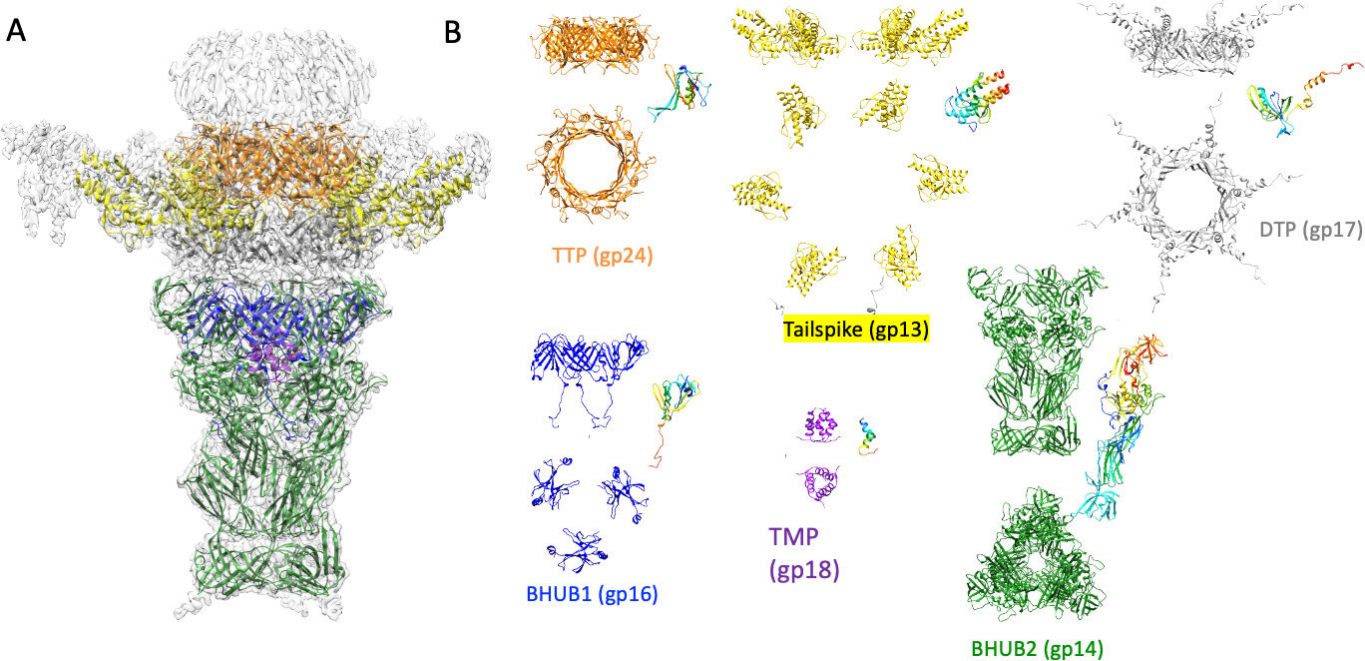
Cryo-EM structure of Sf11 tail apparatus. (**A**) high-resolution, C3 symmetric reconstruction of the tail tip, docked with atomic models. (**B**) Atomic models of individual tail components in side and top-down views. Additionally, a monomer of each protein is shown in a rainbow schematic where the color range extends from the N terminus in blue and the C terminus in red.

We also could not perform a reconstruction of the entire virion, as the tails are very curved and not consistent from particle to particle. However, we performed a focused reconstruction of the tail, including a map where we used symmetrized reconstructions (C3) as well as a completely asymmetric (C1) reconstruction. We resolved the tip of the tail (Fig. 3A) as well as the tail repeating unit (gp24) to 3.2 Å resolution. See Fig. S1 for the cryo-EM processing workflow for tail tip structures. The tail repeating unit forms the length of the siphophage tail as a series of rings (Fig. 3B). Whether the rings stack with true helical symmetry is unclear, as we were unable to analyze the very curvy tails using helical reconstruction approaches. In general, the core of the tail tip of phage Sf11 is highly structurally equivalent to that of phage χ (8). Two proteins within the core are structurally very similar to those in phage χ, including the Baseplate Hub 1 Protein (BH1P, gp16) and Baseplate Hub 2 Protein (BH2P; gp18). See Fig. S3 and S4 for comparisons of these proteins. Additionally, the alpha helical C-terminus of the Tape Measure Protein (TMP; gp18) was observed embedded in the tail tip as has been seen in phages T5 (10), λ (41), 80α (42), χ (8), as well as Bxb1 (43).

**Fig 4:**
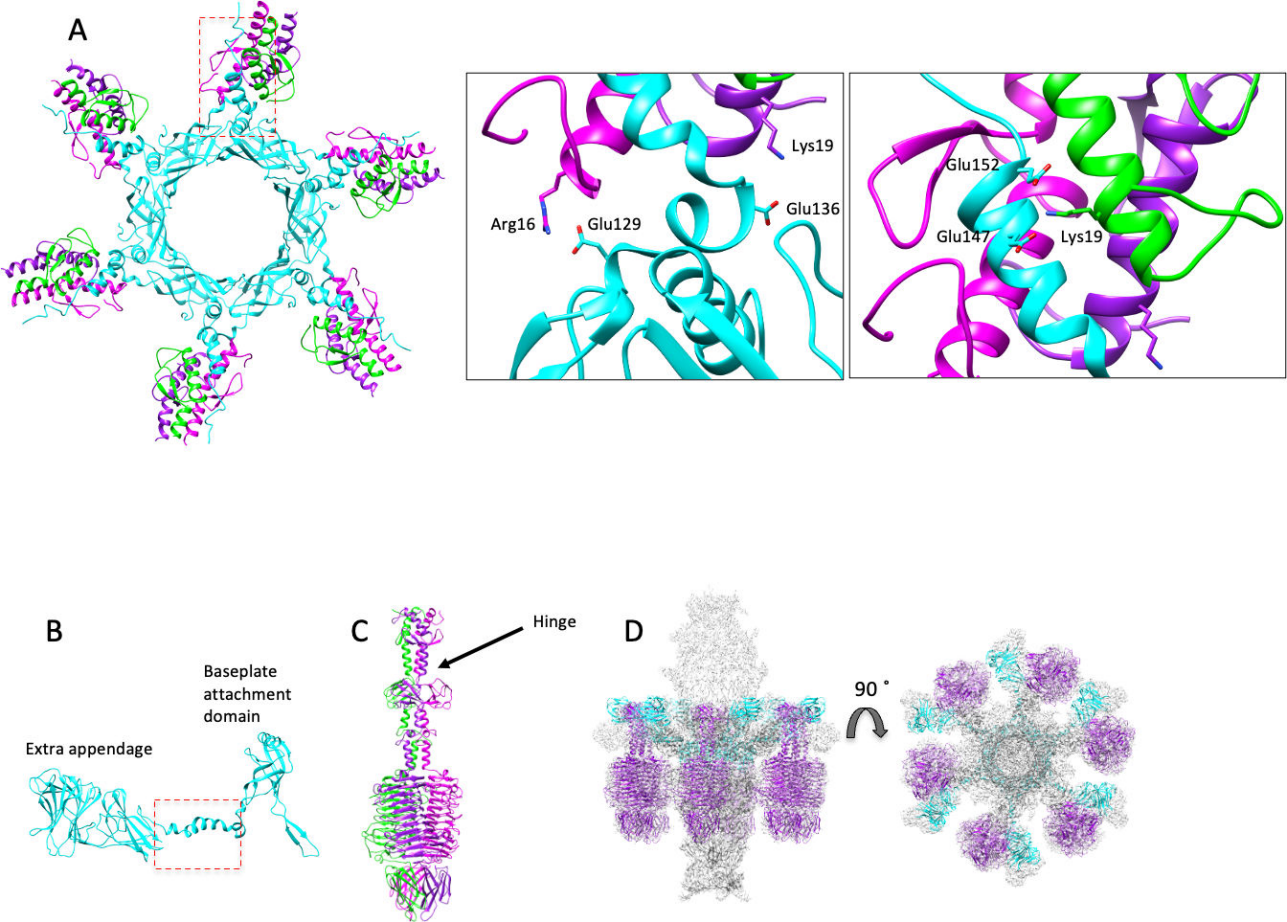
Tailspike and adaptor interactions. (**A**) Atomic models of gp17 (cyan) and gp13 (individual chains of the trimer are shown in green, magenta, and purple) as modeled from the high-resolution cryo-EM data. The left panel shows the overall assembly of six copies of the DTP (gp17) and 18 copies of the tailspike (gp13). The red box highlights the adaptor helical region of gp17 that binds to the tailspikes. Enlarged views in the middle and right panels show some of the specific electrostatic interactions that stabilize the complex. Distances between paired atoms were measured and ranged from 2.3 to 5 Å. Panels** B, C, and D **are Alpha Fold predictions of the full-length proteins. (**B**) Monomeric DTP protein (gp17) showing the large extra appendage. The red box corresponds to the red box in panel **A,** showing the alpha helix of gp17 extending to join the tailspike to the tail. (**C**) Trimer of tailspike protein (gp13) with individual monomers colored in purple, magenta, and green. The arrow points to the “hinge region” starting at residue 162, where the protein bends in the assembled state to attach to the mature virions. (**D**) Six tailspike trimers (gp13, purple) and six adaptor proteins (gp17, cyan) fitted into the cryo-EM density map of the tail tip in the side and top-down views.

A major difference when compared with other siphophages, such as phage χ, in the Sf11 tail tip assembly is the presence of six large appendages (Fig. 1A, arrows). The overall shape of the density map (Fig. 4) is reminiscent of a tailspike appendage, which is an enzymatic protein found in podophages such as *Salmonella* phage P22 (44) and *Shigella* phage Sf6 (45). In Sf6, this is the protein that hydrolyzes *Shigella* lipopolysaccharide (LPS) and determines host range by serotype (19, 46). The presence of a structurally homologous P22-like tail spike in a siphophage has been seen before in a mature virion in the marine phage TW1 (36). How this tail spike appendage was assembled onto the tail machinery could not be determined due to the low resolution of the TW1 structure. In *Salmonella* phage 9NA, a tailspike gene was identified in the genome, and the protein was recombinantly expressed and subsequently crystallized, revealing a P22-like tailspike trimer (47). However, how this was assembled onto the 9NA virion was also not observed. Alternatively, here, we were able to clearly resolve the tailspike attachment to the siphophage tail and identify an adaptor region of DTP (gp17) that is crucial for this protein (gp13) to join onto the virion.

We could not fully model side chains *de novo* for the entire protein length for either DTP (gp17) or tailspike (gp13), as the resolution was a bit lower in this region, likely owing to flexibility (see Fig. S2 for a local resolution map). However, we successfully built atomic models *de novo* for the well-resolved regions of the cryo-EM density map in order to analyze the interaction sites between the two proteins (Fig. 4A). The N-terminal alpha helices of gp13 (residues 4–59) were modeled into the high-resolution tail density map, as well as residues 3–165 of the DTP gp17. The DTP protein has a single helical adaptor region that interacts with all three N-terminal helices from gp13 to create a helical bundle that appears to stabilize the tailspike trimer assembly onto the tail (region highlighted with the red box in Fig. 4A and B). There are several key salt bridges between the four helices. Residue Glu136 in DTP interacts with tailspike Lys19 at a distance of 4.0 Å. A second and third salt bridge forms between two DTP residues, Glu147 and Glu152, with tailspike residue Lys19 at distances of 2.3 Å and 2,7 Å (Fig. 4A). Several longer-range electrostatic interactions (~5–10 Å apart) are also observed along the length of the helical bundle that could stabilize the complex as well. For example, Glu129 on DTP interacts with Arg16 on the tail spike at a distance of around 5.2 Å (Fig. 4A).

We also used AlphaFold to model a monomer of the full-length DTP (gp17) and the full-length tailspike (gp13), as shown in Fig. 4B and C. Like typical P22-like tailspikes (5), the Sf11 gp13 tailspike is a homotrimer. In the Sf11 tail, the DTP protein forms a ring made of six monomer subunits (Fig. 4A and D). Six gp13 trimers join to the gp17 hexameric ring for a total of 18 tailspike protein subunits. The C-terminal region of tailspike trimers binds cell surface receptor(s) such as LPS (46) and OmpC (19) in phages P22 and Sf6. This C-terminal region in Sf11 fits into the density map well without the need for flexible fitting (Fig. 4D). However, the N-terminus of gp13 (residues 1–162) is rigid and elongated in the AlphaFold model (Fig. 4C) and does not dock into the cryo-EM density map of the virion without adjustment. Visual inspection of the AlphaFold models and the cryo-EM density shows that there is likely a flexible “hinge” region around residue 162 that must bend to allow gp13 attachment to the tail.

There is also a large extra appendage of gp17 in the C-terminal domain that extends away from the tail, of currently unknown function (Fig. 4B and D). In order to fully model the C-terminal region, we would need to use flexible fitting to adjust the AlphaFold model. Since the resolution of this region of the map was too poor for accurate flexible fitting, we did not attempt this. The biological purpose of this extra domain is not clear at this time; however, we note this feature is one of the largest DTP proteins resolved in any phage tail that we are aware of. A DALI search of gp17 showed little structural homology to known phage structures, with the top hit being 22% identity and an R.M.S.D. of 16.6 Å and a Z-score of 14.6 for the gp31 sheath protein from *Pseudomonas* phage E217 (48). Interestingly, this protein makes the sheath of a contractile myophage tail and is not functionally related to the DTPs that connect the tails to tips in any known siphophages. The tail tip protein domain is commonly found in tail tips and hubs (Tal and Dit proteins), as well as tail terminator proteins and major tail proteins, suggesting a common evolutionary origin (11, 49).

### Sf11 has an Sf6-like tailspike that utilizes the O-antigen for entry

**Fig 5:**
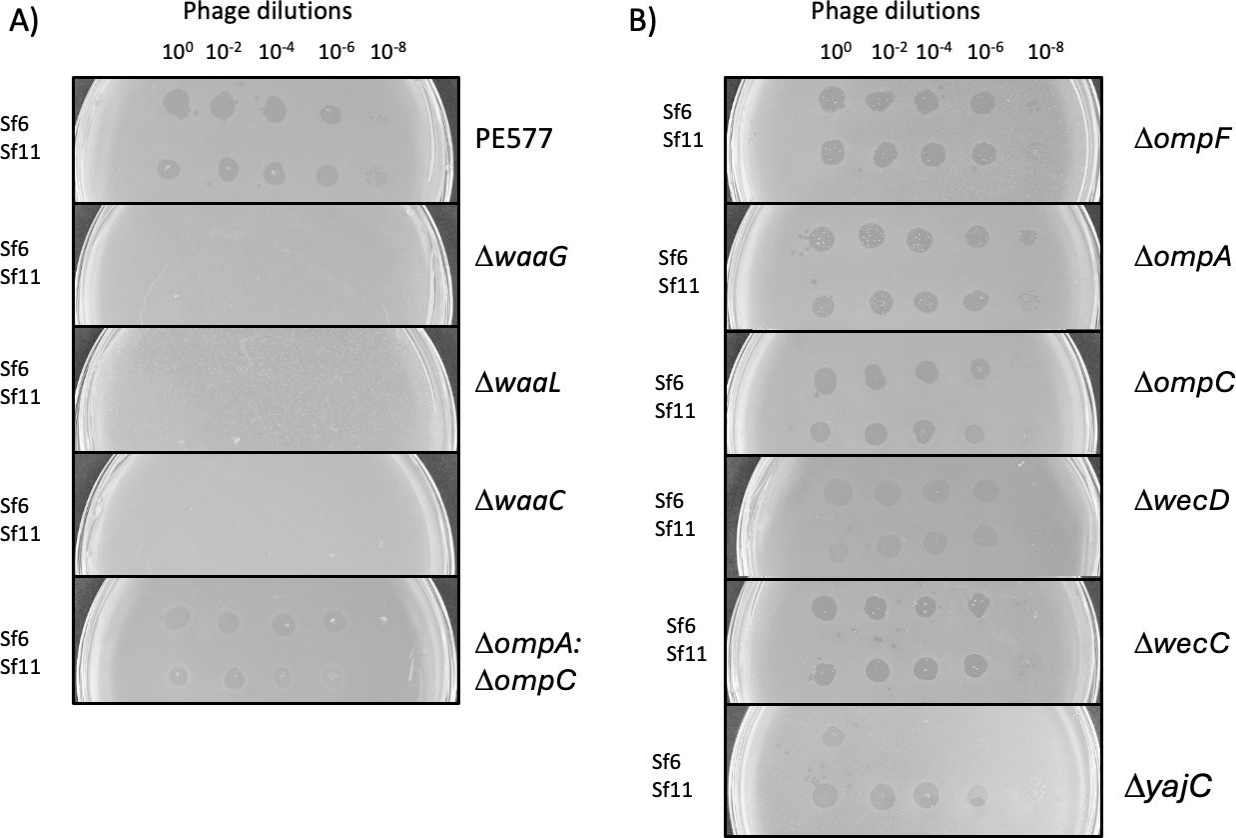
Host range spot tests. Panel (**A**) shows strains that have decreased plating efficiency compared with the permissive host, PE577. Phage Sf6 is used as a positive control. Panel (**B**) shows strains that have no significant change in plating efficiency for Sf11.

Based on the cryo-EM reconstruction of the Sf11 tail, it appeared that Sf11 has a tailspike-like protein. Sf6 utilizes lipopolysaccharide (LPS) as the initial and reversible primary receptor, and outer membrane proteins A or C (Omps) as the secondary, irreversible receptors (16, 17). The enterobacterial common antigen (“ECA”) has been shown to be important for defense against podophages with similar tailspikes, such as *Salmonella* phage P22 (50). Therefore, we tested whether Sf11 uses similar receptors by performing qualitative spot tests on single-gene deletion strains with knockouts of these various surface components (Fig. 5). Productive infections produce plaques, and non-productive infections do not.

Deletions of genes *wecC* and *wecD* (required for ECA synthesis) had no obvious effect on Sf11 plaquing. Next, we tested deletions of *waaC*, *waaL*, or *waaG*, which are truncations to create progressively shorter versions of LPS. All three deletions completely inhibited Sf11 growth, demonstrating that LPS is essential for Sf11 attachment and infection (Fig. 5), and importantly, the full-length molecule is essential. Next, we tested Sf11 for dependence on outer membrane proteins (Omps). Previous work has shown that various Omps can act as receptors for *Shigella* phages, such as OmpA, OmpC, and/or OmpF. Individual *ompF*, *ompA,* or *ompC* knockouts had no effect on Sf11 plaque formation. Taken together, these results indicate that these outer membrane proteins are not essential for Sf11 infection. Lastly, the inner membrane protein YajC was found to be essential for Sf6 as previously shown (25), but was not essential for Sf11 entry.

### Sf11 entry can occur anywhere on the cell surface

**Fig 6:**
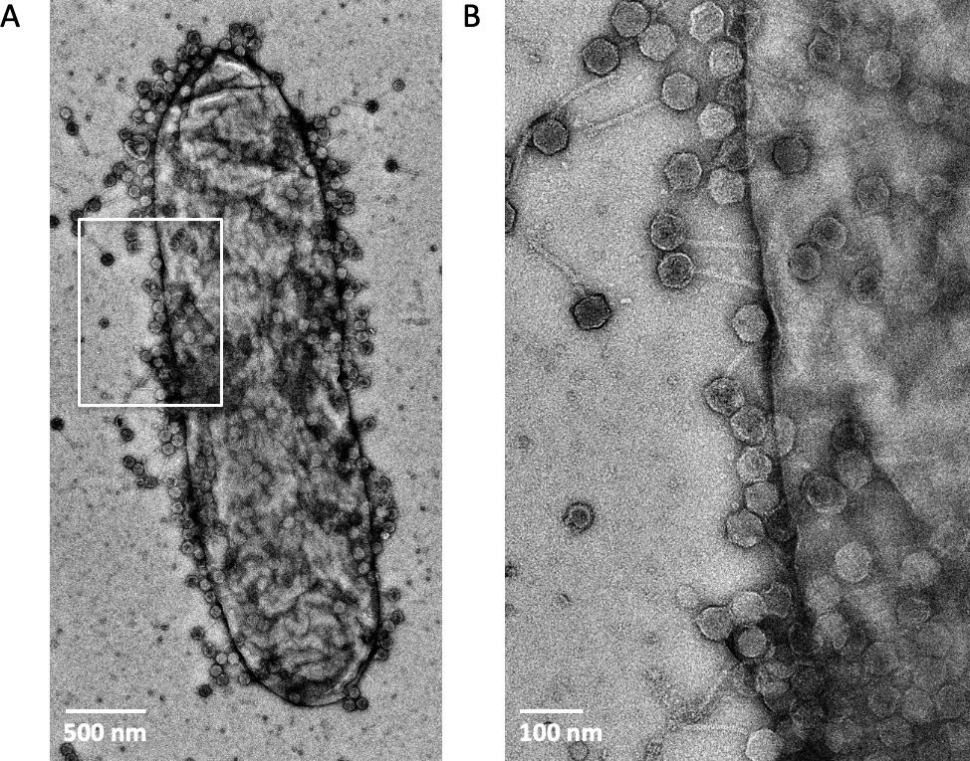
Representative micrograph of negatively stained Sf11 attached to PE577 (serotype Y *S*. *flexneri*). (**A**) A lower magnification view of an individual cell showing phage decorating the entire surface. (**B**) Enlarged view of the area highlighted with the white box shown in panel **A**.

Some siphophages are known to be flagellotropic, meaning they bind to the bacterial flagella as a primary receptor and then bind to LPS as a secondary receptor (e.g., phage χ) (51, 52). In this model, the siphophages “walk” down the flagella in a directional manner. By contrast, others bind preferentially to very specific membrane transporters, which are localized to specific areas of the cell surface. For a classical example, phage λ binds to a sole proteinaceous receptor, LamB, which is preferentially localized to the cell poles (53). Since *Shigella* are non-flagellated (54), and the putative primary receptor is LPS based on our host range studies (Fig. 5), we hypothesized that Sf11 could bind anywhere on the *Shigella flexneri* cell surface and would not likely demonstrate preferential binding. To test this hypothesis, we imaged phage bound to host cells using electron microscopy of negatively stained samples (Fig. 6). After a relatively short incubation period of 30 min at 22°C, we saw phages bound all over the surface of *S. flexneri* PE577 cells, indicating no specific binding sites for Sf11 (Fig. 6). This is similar behavior to *Shigella* podophage Sf6, which has a similar tailspike protein and also does not demonstrate preferential binding to specific cell surface features (16).

### Summary

Traditionally, siphophages have relatively few types of tail tip morphologies, and the DTP often serves as an attachment site for critical components of the infection machinery. Typically, this protein forms a hexameric ring and represents a baseplate docking site (1, 3), with the C-terminal domain displaying variability in terms of structural moieties providing a means for different mechanisms of host adsorption (49). This has been observed in phage T5 (55), where the DTP serves to attach the side tail fibers, and in phage p2, it serves as an attachment site for the receptor binding protein (RBP) (56). In a recent structure of phage χ, extensions on the DTP were proposed to serve an alternative function. Sonani et al. suggested that the ring formed by DTP serves to reduce the complexity of a symmetry mismatch between the C6 symmetry of the tail and the C3 symmetry of the tip (8). The extension of the C-terminal domain of gp17 serves both functions in *Shigella* phage Sf11, simultaneously bridging symmetry mismatches between the tail and tip and providing a docking site for the LPS-hydrolyzing tailspike gp13. Additionally, comparing the virion shape while in solution (Fig. 1A) with that attached to cells (Fig. 6) reveals that upon cell attachment, the virion tails stiffen and no longer appear flexible. It is likely that once the tail spike appendage docks to the LPS receptor and the tail tip interacts with the membrane, a signal is transmitted along the length of the tail, inducing conformational changes and subsequent rigidity to allow the dsDNA genome to transmit along the length of the tail into the host. Atomic details of this mechanism remain to be determined and will be the focus of future studies.

Why would siphophage Sf11 have a podophage tail spike, and how did it acquire this appendage? It appears that many *Shigella* phages utilize tailspike proteins in their tails. This is well documented for phage Sf6 (46), which has a tail machine very similar to *E. coli* phage CUS-3 and *Salmonella* phage P22 (57). Tail spikes have also been reported in other *Shigella* podophages with drastically different morphologies. Examples include phage HRP29, which has a hybrid tail that is a mix between phages T7 and Sf6 (25), as well as phage Moo19, which is an N4-like phage with an entirely different tail baseplate (24). The tailspike is important for host recognition in these *Shigella* phages, and they can evolve to infect new hosts through point mutations in this protein (19). Since the vast majority of known *Shigella* phages come from phage hunting studies (12, 21–23) using similar “bait bacteria,” it is possible we are selecting for phages that preferentially use this receptor and have an overabundance of this structure in our library. However, the fact that this same structural unit is found in phages with such diverse morphologies suggests a strong evolutionary relationship between these phages, as conserved structures found in nature often highlight their biological importance. Proteins involved in the receptor binding process can be conserved across broad groups of phages. This phenomenon of shared receptor protein structures between siphophages and podophages has been observed in other systems, such as *Staphylococcal* phages (9, 58, 59). Since horizontal gene transfer is a common evolutionary mechanism among phages, tail component sharing may be facilitated by horizontal acquisition across highly diverse phages.

Adaptor domains that allow for tailspike protein addition to diverse tail structures may be a way to allow for a universal, and “generalist” strategy of using tailspikes to aid in *Shigella* phage cell entry. This could be exploited to create designer phages with different tail appendages for use in therapeutic and/or industrial applications.

## MATERIALS AND METHODS

### Phage isolation, purification, and amplification

Sf11 stocks were grown from a single plaque in a 30 mL preparation using our standard approaches and yielded a working stock at 4 × 10^11^ plaque-forming units per mL (PFU/mL). This high-titer working stock was used to seed a larger, 500 mL prep at a multiplicity of infection (MOI) of 0.1 in LB with *S. flexneri* PE577 (serotype Y) for 2 h, followed by spiking the culture with additional cells. The prep was grown for a total of 4 h at 37°C while shaking at 200 rpm. The lysate was centrifuged at 4°C for 30 min at 8,000 × *g* to remove debris, and the supernatant was spun at 4°C for 120 min at 20,000 × *g*. The phage pellet was then resuspended by overnight nutation at 4°C with 4 mL of phage dilution buffer (10 mM Tris, pH 7.6, 10 mM MgCl_2_) for a final titer of ~1.5 × 10^12^ PFU/mL.

### Transmission electron microscopy and three-dimensional image reconstruction

A small aliquot (~5 µL) of phage was added to Quantifoil R2/2 grids that were glow-discharged using a Pelco EasiGlow glow discharge apparatus and frozen with a Vitrobot Mark IV with a blot force of 1, blot time of 5 s, at 4°C and 100% humidity. Cryo-EM data were collected at the University of Michigan Cryo-EM facility using a Titan Krios equipped with a K3 direct electron detector and operating at 300 keV with a post-column GIF (20 eV slit width). Micrographs were collected at 105,000× magnification (0.834 Å/pixel) by recording 50 frames over 1.93 s for a total dose of 50.45 e-/Å2. Data processing was carried out using Cryosparc v4.6.0 (60). The micrographs were first motion corrected using Patch Motion correction, followed by CTF estimation using Patch CTF estimation, and particles were picked using template picker. For capsid, a total of 43,242 particles were used for 3D refinement (Icosahedral symmetry), and for tail, a total of 35,748 particles were used for 3D refinement (C3 symmetry). The overall map resolutions were estimated based on the gold-standard Fourier shell correlation (FSC 0.143) (61). The final maps were deposited into the Electron Microscopy Data Bank (EMD-70309, EMD-70310, and EMD-72999; see Table 2). We generated initial models using ModelAngelo (57), using a combination of both sequence and non-sequence modes. Refinement was carried out using Phenix (58), and model adjustments were carried out in COOT (59). Models were deposited into the Protein Data Bank (PDB ID: 9OCB, 9OCC, and 9YIN; see Table 2)

**TABLE 2:** Map and protein model statistics

Map EMD identifier PDB identifier	Sf11 capsid EMD-70309 9OCB	Sf11 tail EMD-70310 9OCC	Sf11 portal EMD-72999 9YIN
Data collection and processing			
Magnification	105,000	105,000	105,000
Voltage (kV)	300	300	300
Electron exposure (e–/Å^2^)	50.45	50.45	50.45
Defocus range (μm)	-0.5 to - 2.5	-0.8 to - 3.0	-0.8 to - 3.0
Pixel size (Å)	0.834	0.834	0.834
Symmetry imposed	I1	C3	C12
Number of micrographs	17,972	17,972	17,972
Number of particles	43,242	35,748	20,482
Map resolution (Å) FSC_0.143_	3.4	3.2	2.4
Refinement			
R.m.s. deviations			
Bond lengths (Å)	0.007	0.007	0.003
Bond angles (°)	0.997	0.776	0.536
Validation			
MolProbity score	2.23	2.18	1.49
Clashscore	15.22	9.69	3.94
Poor rotamers (%)	1.74	2.25	1.59
Ramachandran plot			
Favored (%)	94.84	93.98	97.11
Allowed (%)	4.40	5.64	2.89
Disallowed (%)	0.75	0.38	0.00
CC (volume)	0.80	0.89	0.91
d FSC model (0.5) (Å)	3.5	3.4	2.7

### Host range and efficiency of plating

Initial host range results were performed by combining bacterial cells in a double agar overlay method. Receptor binding assays were done by spot tests. Once agar containing each bacterial host solidified, 2 µL of phage stock and subsequent serial dilutions were applied to the top of the agar and left to dry before incubating overnight. Hosts with a positive result showed a cleared spot the next day, whereas hosts that produced a negative result had no inhibited cell growth. All spot tests were done with biological replicates (at least three plates per strain).

### Negative staining of phage attachment to hosts

An equal volume of PE577 cells grown overnight in LB at roughly ~10^8^ cells/mL and phage Sf11 stock at ~1 × 10^11^ phage per mL were incubated for 30 min at RT (~22°C). A small aliquot of 5 µL was deposited onto continuous carbon copper grids (TedPella) and stained with 1% uranyl acetate. Micrographs were imaged using a Talos Arctica and a Ceta camera at a lower magnification (13,500×; pixel size of 1.07 nm) and at a higher magnification of 45,000× (pixel size of 3.2 Å)

## Data Availability

All data needed to evaluate the conclusions in the paper are present in the paper. See Table 2 for the accession numbers for both the cryo-EM density maps (deposited to the EMDB) and corresponding atomic models (deposited to the PDB).

## SUPPLEMENTAL MATERIAL

Supplemental figures jvi.01367-25-s0001.docx Figures S1 to S4.

ASM does not own the copyrights to Supplemental Material that may be linked to, or accessed through, an article. The authors have granted ASM a non-exclusive, world-wide license to publish the Supplemental Material files. Please contact the corresponding author directly for reuse.
