# Supplementary material for: The Shigella siphophage Sf11 tail structure and host attachment mechanism
Source: J Virol. 2025 Nov 19;99(12):e01367-25. doi: 10.1128/jvi.01367-25 (PMC12724387; doi:10.1128/jvi.01367-25)

Supplementary Materials

Figure S1: Cryo-EM processing pipeline for Sf11 capsid and tail tip.


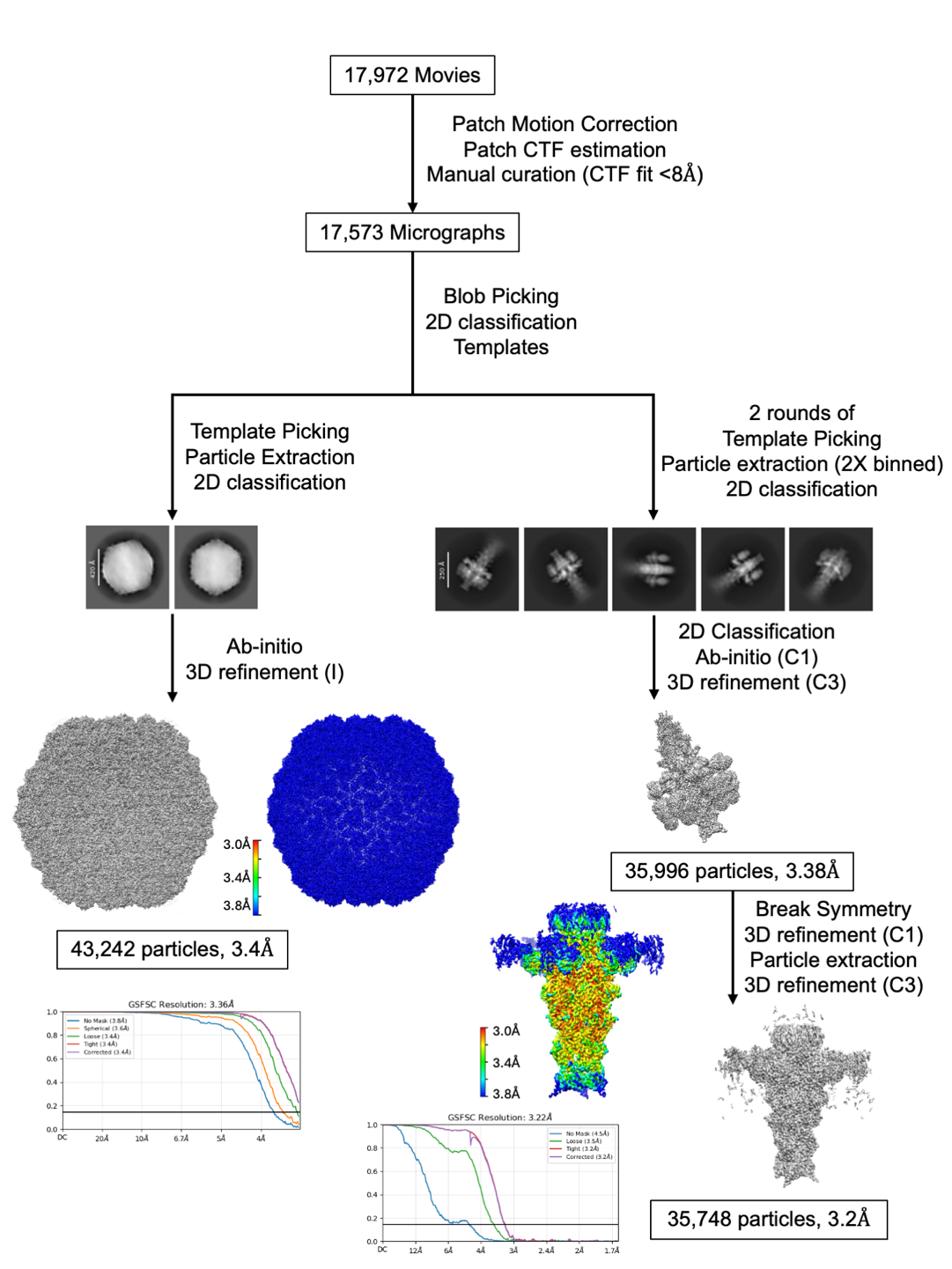


Figure S2: Cryo-EM processing pipeline for Sf11 portal.


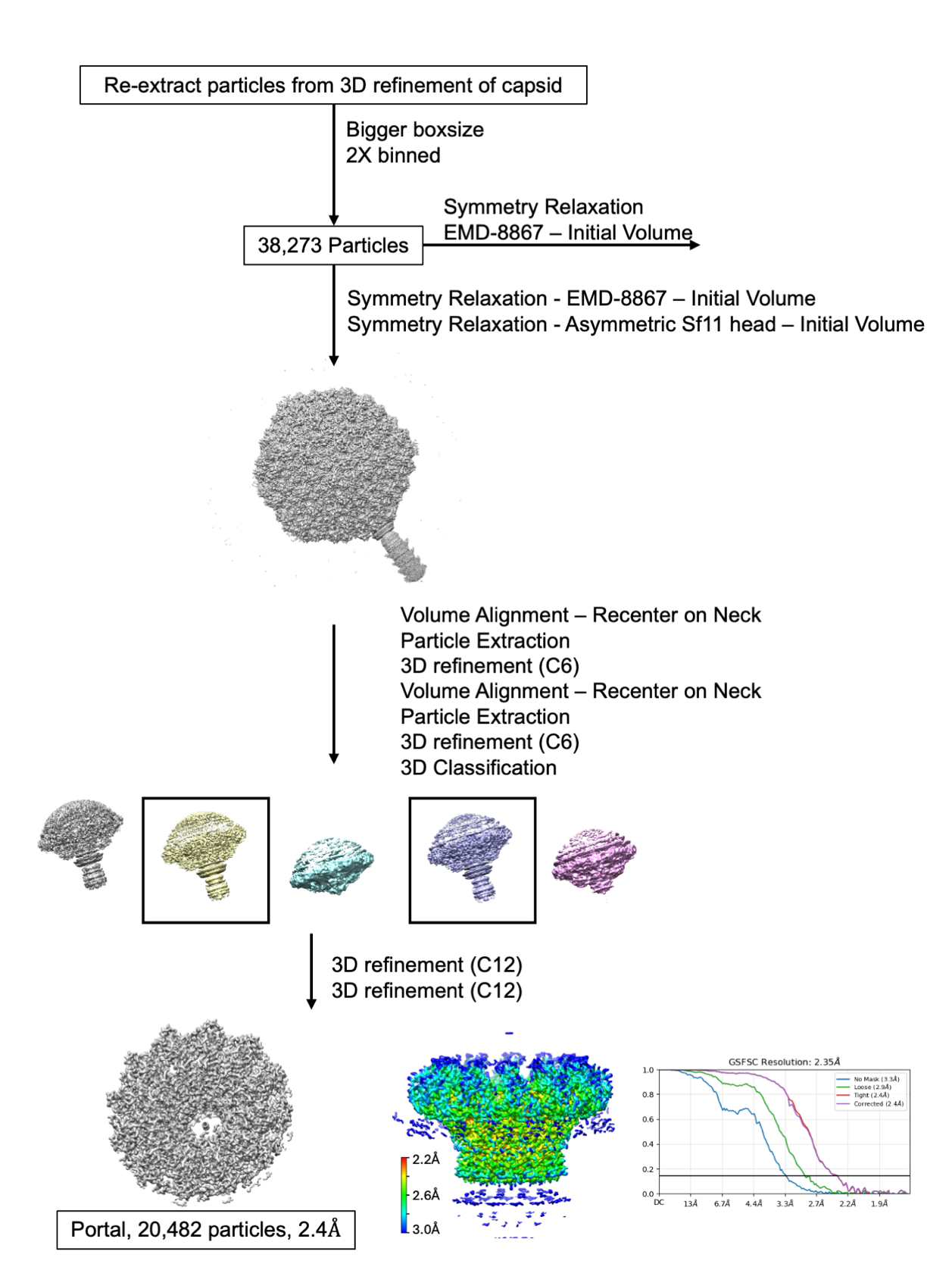


Figure S3: Structure comparison between Sf11 and phage χ BHUB2 proteins. Left is a aside by side comparison of BHUB2 proteins from Sf11 and phage χ in a rainbow color scheme where the N-terminus is blue and the C-terminus is red. Right is an overlay of the two structures, aligned using UCSF Chimera matchmaker tool. Phage χ has a long flexible region, midprotein, that results in the BHUB2 protein being more twisted in the mature phage χ tail tip when compared with Sf11, resulting in misalignment of half the protein. In order to account for this when calculating structural similarity, we independently fitted the N-terminal and C-terminal halves of phage χ’s BHUB2 to Sf11. With the linker removed, phage χ’s BHUB 2 residues 2-413 aligned well and had an R.M.S.D. of 1.4 Å. Additionally, residues 414-770 aligned well with an R.M.S.D of 1Å.


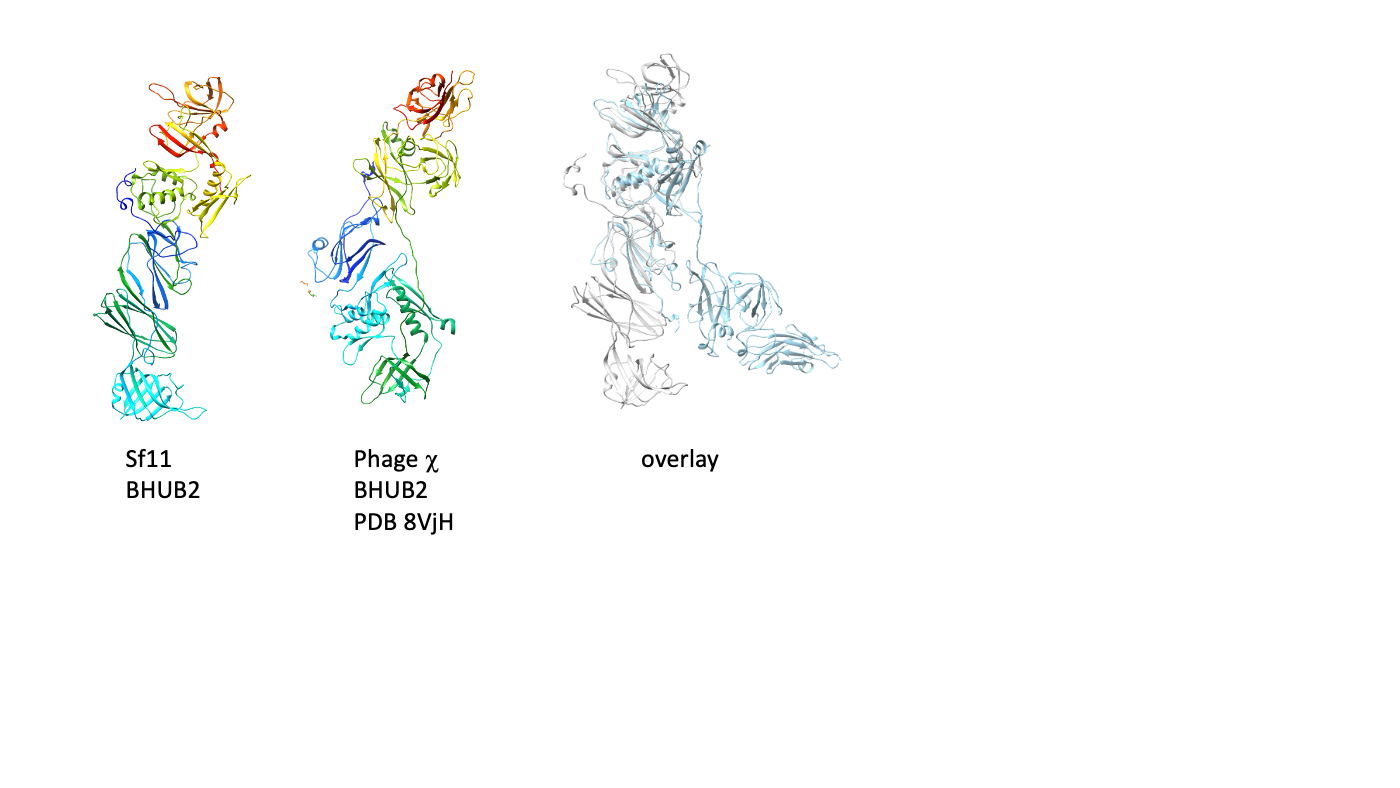


Figure S4: Structure comparison between Sf11 and phage χ BHUB1 proteins. Left is a aside by side comparison of BHUB2 proteins from Sf11 and phage χ in a rainbow color scheme where the N-terminus is blue and the C-terminus is red. Right is an overlay of the two structures, aligned using UCSF Chimera matchmaker tool. 143 residues aligned to an R.M.S.D. of 1 Å. Phage χ’s BHUB1 is a longer protein with an extra domain (yellow in the middle panel) compared with Sf11’s BHUB1.


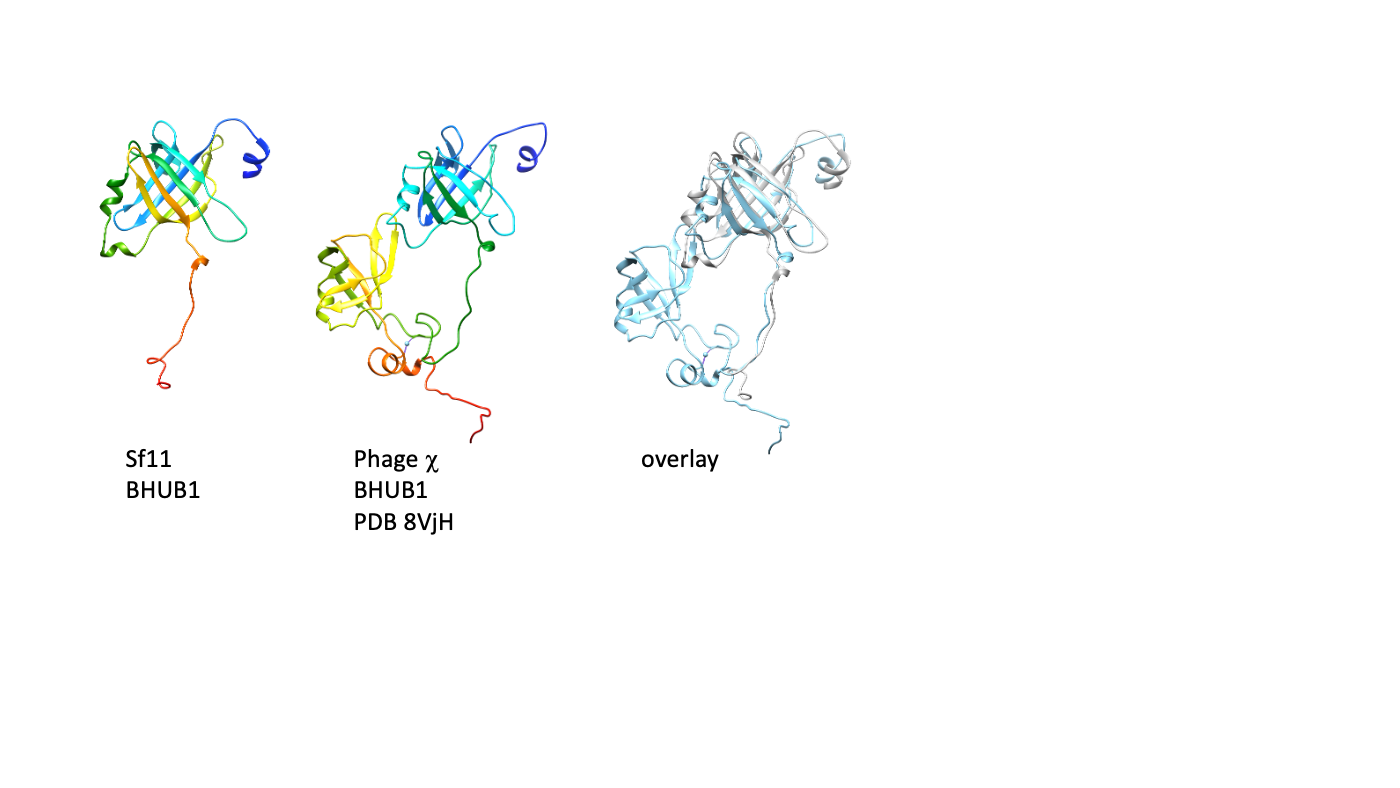

Supplement: Supplemental figures — Figures S1 to S4. [file jvi.01367-25-s0001.docx]
